# Cohort profile: a nationwide retrospective cohort of mortality in people living with HIV in Korea, 1985-2020

**DOI:** 10.4178/epih.e2025002

**Published:** 2025-01-02

**Authors:** Taeyoung Kim, Yoonhee Jung, Koun Kim, Jung Wan Park, Jeonghee Yu, Sung-il Cho

**Affiliations:** 1 Division of HIV/AIDS Prevention, Korea Disease Control and Prevention Agency, Cheongju, Korea; 2 Graduate School of Public Health and Institute of Health and Environment, Seoul National University, Seoul, Korea; 3 Division of Infectious Diseases, Department of Internal Medicine, Soonchunhyang University, Cheonan, Korea

**Keywords:** Human immunodeficiency virus, Acquired immunodeficiency syndrome, Cohort studies, Mortality

## Abstract

The increasing number of people living with human immunodeficiency virus (HIV) in Korea has prompted interest in using the national surveillance system as a database for studying their health. To investigate the relationships between socio-demographic and epidemiological characteristics and mortality rates, a nationwide retrospective cohort was formed by integrating surveillance data with the Cause of Death Statistics from Statistics Korea. This integration included incidence reports, epidemiological investigations, and death reports from the surveillance data, enriched with detailed mortality information from the Cause of Death data. The cohort comprised 17,199 adult Korean individuals diagnosed with HIV infection from 1985 to 2020. By the end of 2020, 2,721 of these individuals were confirmed deceased. The sex ratio of the study participants was 14.3:1.0 (male to female), with 78.2% being under 50 years old at the time of diagnosis. Sexual contact was identified as the primary transmission route, accounting for 75.7% of cases. HIV disease emerged as the predominant cause of death, representing two-thirds (1,817 of 2,721) of the fatalities, followed by injuries and trauma, malignancies, and cardiovascular diseases. Recommendations for further cohort studies may be submitted to the Korea Disease Control and Prevention Agency.

## INTRODUCTION

The human immunodeficiency virus (HIV) and acquired immune deficiency syndrome (AIDS) pandemic has had a profound health and socioeconomic impact on societies worldwide since the 1980s. It continues to be a major public health threat, with over 42.3 million deaths globally as of 2023, and approximately 39.9 million people living with HIV (PLHIV) by the end of the year [1]. However, antiretroviral treatment (ART) has somewhat alleviated the burden for PLHIV. In 2023, it was estimated that 30.7 million out of the 39.9 million PLHIV were receiving ART [2]. Furthermore, reports indicate that ART has significantly increased their life expectancies by several decades [3-5].

In Korea, the number of PLHIV has been rising over the decades since the first laboratory-confirmed case of HIV infection in 1985, mirroring a global trend, according to the Korea Disease Control and Prevention Agency (KDCA) [6]. Since 2013, the annual number of reported PLHIV has consistently surpassed 1,000, with the exception of 2021. Additionally, the number of reported deaths among PLHIV has exceeded 100 each year. Of the 19,745 reported cases of PLHIV with Korean nationality, 16,467 were still alive at the end of 2023. According to a recent report by the Organization for Economic Cooperation and Development on national HIV epidemics in Asia and the Pacific in 2021 [7], both the annual incidence and prevalence of HIV infection in Korea are considered lower than in other countries.

With biomedical advancements, including improvements in ART, PLHIV are experiencing longer lifespans. Studies in Korea have investigated whether survival rates among PLHIV have improved. These studies indicate that factors such as age, biological sex, year of diagnosis, year of ART initiation, initial blood CD4+ T cell count (often used as an indicator of the time from infection to diagnosis), and comorbidities are associated with mortality [8-12]. Research efforts continue under the Korea HIV/AIDS Cohort Study, a national-level, hospital-based prospective cohort that had enrolled 1,438 PLHIV by 2016 [13,14]. However, studies conducted in clinical settings have not accounted for mortalities outside of hospitals. A previous analysis of 5,313 PLHIV, based on national HIV/AIDS surveillance, lacked data on causes of death [9]. Another national-level study examined the clinical characteristics and causes of death among 13,919 PLHIV using National Health Insurance databases and mortality statistics. Despite these efforts, certain gaps remain that necessitate further exploration of PLHIV’s mortality rates. Notably, PLHIV who were tested outside of medical facilities and did not receive treatment may have been excluded from the study, as it relied on health insurance data. Additionally, the study focused on clinical characteristics but did not address epidemiological characteristics, which could also provide valuable insights into their health.

In this context, our study aimed to explore the associations between socio-demographic and epidemiological characteristics and mortality rates among PLHIV. By utilizing national surveillance data and mortality statistics, we sought to identify populations at higher risk of mortality.

## COHORT DESCRIPTION

### The national HIV/AIDS surveillance system in Korea

**Figure 1.**
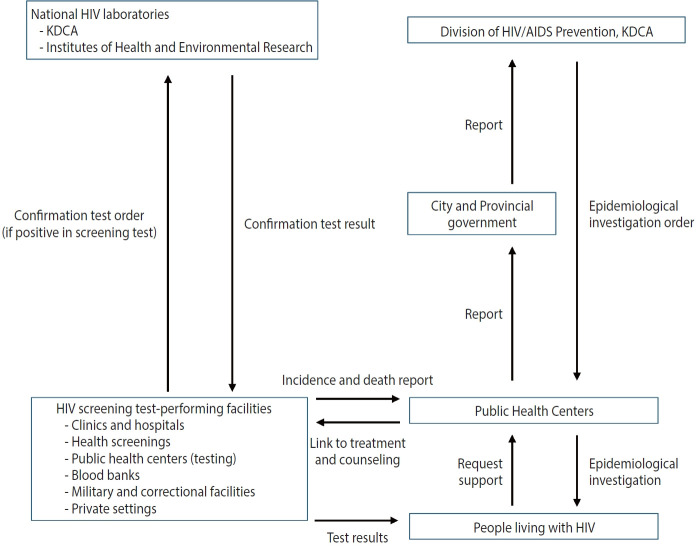
Overview of the national HIV/AIDS surveillance system. HIV, human immunodeficiency virus; AIDS, acquired immune deficiency syndrome; KDCA, Korea Disease Control and Prevention Agency.

Since the enactment of the Prevention of Acquired Immunodeficiency Syndrome Act in 1987, a national surveillance system has been established to monitor HIV infection across the country. This system mandates that physicians, clinics, hospitals, and facilities like blood donation centers conducting HIV laboratory tests report all recognized cases of HIV infection (Figure 1) [15]. Following these reports, public health officials conduct epidemiological investigations. These investigations include recording demographic characteristics such as age, biological sex, marital status, and occupation (Table 1). Epidemiological data, including high-risk behaviors, suspected transmission routes, reasons for undergoing HIV testing, and partners’ test results, are collected on a self-report basis. Clinical data, such as blood CD4+ T cell counts, viral loads, and AIDS diagnoses, are also reported when available. Additionally, any deaths among PLHIV are reported upon recognition.

### Cohort data curation

**Figure 2.**
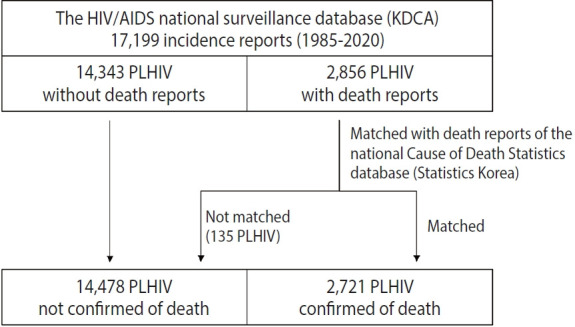
Cohort data curation. HIV, human immunodeficiency virus; AIDS, acquired immune deficiency syndrome; PLHIV, people living with HIV; KDCA, Korea Disease Control and Prevention Agency.

In our study, we obtained national surveillance data that included demographic and epidemiologic information of individuals, as well as 17,199 incident reports, of which 2,856 were death reports. To verify the accuracy of these death reports, we merged a subset of 2,856 individuals’ death reports from the surveillance data with the Cause of Death Statistics data published by Statistics Korea (Figure 2) [16]. Of the 2,856 death reports analyzed, 2,721 individuals were confirmed deceased according to the Cause of Death Statistics. For these matched individuals, we accessed additional data from the Cause of Death Statistics, including the cause, time, and location of death, as well as marital status, highest educational level, and occupation. However, 135 death reports did not match; these were excluded from the study as invalid. Of these, 87 reports lacked a manually recorded cause of death, and the categorization in the remaining reports was inconsistent.

### Study subjects

Individuals of Korean nationality diagnosed with HIV infection between 1985 and 2020 were included in this study. Those under 18 years old at the time of their laboratory-confirmed diagnosis were excluded due to ethical concerns. Non-Korean nationals reported to have HIV infection were also excluded, as they were more likely to have been initially diagnosed outside of Korea, and the likelihood of their deaths being reported while in Korea could not be reliably compared with that of Korean nationals. Additionally, some individuals were anonymously reported, as allowed under the Prevention of Acquired Immunodeficiency Syndrome Act; these cases were excluded because their demographic and epidemiological data were unobtainable.

### Reports and investigations

When individuals were diagnosed with HIV infection, public health authorities received reports containing socio-demographic information and details of the HIV tests. The locations for HIV screening tests, which included enzyme-linked immunosorbent assays and rapid antigen tests, were classified into 8 categories: medical facilities, public health centers, blood banks, military facilities, correctional facilities, quarantine areas, private settings, and others. Tests were administered in medical facilities and public health centers, driven by medical requirements or individual preferences. Additionally, screenings were performed prior to blood donations to prevent the transmission of infection through contaminated blood. Upon entry, screenings were also mandatory in military and correctional facilities, as well as at quarantine checkpoints. Furthermore, tests were available in private settings, such as government-supported testing centers. The date of laboratory confirmation was defined as the day on which individuals were confirmed HIV-positive through an HIV western blot test or a nucleic acid test, including polymerase chain reaction tests, conducted by the national laboratories of KDCA or regional Institutes of Health and Environmental Research.

Following the reports, public health officials conducted epidemiological investigations either in person or by telephone, based on individuals’ responses. They gathered information on the reasons for testing, experiences of high-risk HIV exposure, and self-suspected transmission routes. The reasons for testing were divided into 9 categories: general screening, illness, voluntary testing, pre-hospitalization or surgery screening, partner notification, mandatory routine testing, blood donation, others, and not available. General screening encompassed various medical screenings, including national health programs and private checkups. When a physician ordered an HIV test to assist in differential diagnosis, the reason was categorized as illness. Tests initiated by the individuals themselves were classified as voluntary. Some tests were precautionary measures before hospitalizations or surgeries. Partner notification referred to test recommendations from sexual partners or individuals engaged in risky behaviors. Mandatory routine testing was reported for workers in professions with a high risk of HIV, such as sex workers. Testing related to blood donations was specifically categorized under blood donation. Based on the information about high-risk experiences and self-suspected transmission routes, KDCA officials categorized the suspected transmission routes into 6 groups: opposite-sex sexual contact, samesex sexual contact, blood transfusion, vertical transmission, injection drug use, and not available. If the transmission routes could not be definitively categorized due to logical inconsistencies, ambiguities, or if contact with the individual was not possible for further investigation, they were assigned to the “not available” category.

Death reports were submitted to the national surveillance system following the death of either newly diagnosed individuals or those previously reported. Detailed information about these deaths was sourced from the Cause of Death Statistics database. This database includes mortality reports from physicians and medical institutions, as required by the Medical Service Act [17], and also features socio-demographic data verified by government officials. The causes of death were organized into 237 categories according to the 8th revision of the Korean Standard Classification of Diseases (KCD-8) [18].

### Ethics statement

This study protocol received approval from the Institutional Review Board (IRB) of KDCA (IRB No. 2022-09-06-PE-A). The IRB waived the requirement for obtaining individual consent since data collection involved the national surveillance and the Cause of Death Statistics databases, which are governed by relevant laws. Additionally, obtaining retrospective consent was deemed impractical due to the absence of personal contact information. Data merging was restricted to individuals reported deceased in the national HIV/AIDS surveillance system, in compliance with the Personal Information Protection Act, to maintain the confidentiality of surviving individuals’ personal information [19]. All personal identification details, including resident registration numbers, were removed during the data merging process.

## KEY FINDINGS

The number of newly diagnosed PLHIV increased over time, reaching a peak between 2011 and 2015, followed by a slight decline from 2016 to 2020 (Table 2). Of the 17,199 PLHIV reported, 78.2% were diagnosed before the age of 50, an age range typically associated with sexual activity. Case fatality rates were generally higher among those diagnosed in earlier years or at older ages. Among the reported cases, 93.5% were biologically male, although over a thousand female cases were reported. There was no significant difference in the case fatality rates between the male and female populations (p= 0.61, chi-square test). Of the 17,199 PLHIV, 56.8% were unmarried, and 24.7% were tested for HIV due to illness (Table 3). The majority of PLHIV were tested in medical facilities (66.3%) or public health centers (23.4%). Among those with known transmission routes, 56.8% (7,427 of 13,066) were suspected to have contracted the virus through heterosexual contact, and 42.8% (5,598 of 13,066) through homosexual contact. There were 32 cases of infection via blood transfusion, with none reported in the 2010s. Additionally, there were 9 reported cases of infection through injection drug use. No cases of vertical transmission were reported among adult PLHIV.

**Figure 3.**
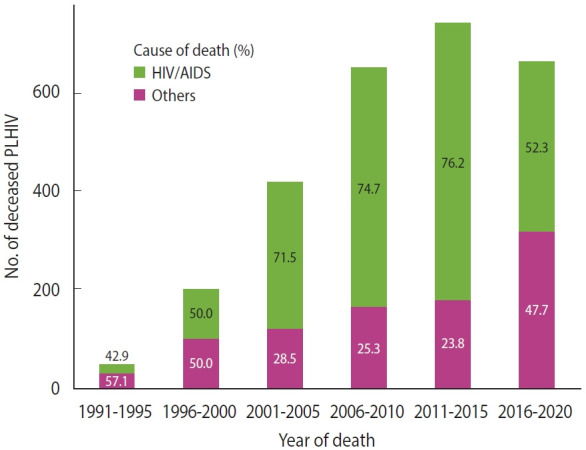
HIV/AIDS-related and non-HIV deaths of PLHIV, reported in 1985-2020. Causes of death other than ‘HIV disease (B20-B24)’ were sorted to ‘Others.’ HIV, human immunodeficiency virus; AIDS, acquired immune deficiency syndrome; PLHIV, people living with HIV.

HIV disease was the leading cause of death, accounting for 66.8% of the fatalities among the 2,721 PLHIV with confirmed death reports (Table 4). The incidence of deaths attributed to HIV/AIDS reached its peak between 2011 and 2015, followed by a significant decline from 2016 to 2020 (Figure 3). In contrast, deaths from causes other than HIV/AIDS continued to increase. Among the top 20 causes of death excluding HIV disease, 5 were related to external factors, such as trauma and substance use. Pneumonia, tuberculosis, liver and lung malignancies, and acute myocardial infarction were also among the 10 most common causes of death among PLHIV.

## STRENGTHS AND WEAKNESSES

Numerous studies globally have aimed to analyze the characteristics of PLHIV using integrated data. Many of these studies have concentrated on assessing the effectiveness of ART and the survival rates from AIDS-related illnesses, involving PLHIV who are either ART-naïve or have just initiated ART treatment at medical centers. Additionally, some research has investigated the correlations between HIV-related epidemiological characteristics and the mortality rates of PLHIV [20,21]. In Korea, the multicenter-based prospective HIV/AIDS Cohort Study was established to thoroughly examine the clinical, epidemiological, and mortality characteristics of PLHIV [13]. Furthermore, single-center cohort studies have been conducted to identify the risk factors associated with the mortality of PLHIV and the causes of these deaths [8,10,11,22].

Our cohort was established using the national disease surveillance system, which is among the most comprehensive nationwide databases available. In contrast to studies that rely on data from medical centers, our cohort includes participants irrespective of their treatment status and incorporates mortality data from Statistics Korea, encompassing all causes of death. This study design enables us to track the mortality of all reported PLHIV, including those deaths that occur outside of medical facilities. Therefore, research based on this cohort can offer a detailed overview of the national HIV/AIDS epidemic. Additionally, the mortality data for PLHIV is meticulously organized and dependable, as the Cause of Death database is nationally recognized and subject to rigorous standardization and quality control processes.

Studies based on this cohort may have some limitations and should therefore be interpreted with caution. First, similar to other observational studies on HIV/AIDS, this cohort did not include undiagnosed PLHIV, who are likely at a higher risk of illness and death. A recent study estimated that 37.5% of PLHIV in Korea might not have been aware of their HIV-infected status by 2020 [23]. Additionally, there was a discrepancy between the number of HIV-attributed deaths (codes B20-B24 in the KCD-8) reported in the cohort (n= 1,817) and the number reported in the publicly available database from Statistics Korea (n= 1,952) [24]. This difference raises the possibility that some PLHIV diagnoses or deaths were not reported to the national surveillance system, although delayed reports to Statistics Korea might have also contributed to the discrepancy.

Anonymously reported PLHIV, who either opted not to disclose their personal information or had been previously reported by other physicians, were excluded from the cohort. It was impossible to estimate their number and characteristics because the anonymous report form lacked identifying and epidemiological details, and no epidemiological investigation was conducted on these reports. Given that both their number and mortality risk remain unknown, selection bias must be considered when generalizing the findings from this cohort.

Reports of PLHIV who are not of Korean nationality were also excluded from the cohort due to challenges in tracking their survival based on death reports. Given that the proportion of newly infected PLHIV who are non-Korean nationals has recently risen to 25% [6], this exclusion could introduce further bias when attempting to infer the characteristics of the general PLHIV population in Korea.

In addition, the lack of clinical data significantly limits the cohort, as details about ART and clinical conditions could be key predictors of mortality among PLHIV and assist in validating causes of death reported in the records. The integration of national surveillance data with other data sources has been restricted to safeguard the confidentiality of PLHIV. Nonetheless, future cohort studies might explore the inclusion of additional data sources, taking into account legal and ethical considerations.

Lastly, the information obtained from epidemiological investigations was based on self-reported responses from PLHIV; some types of information could not be validated with sources other than self-reports. Therefore, these data should be interpreted with consideration of their collection process and the external circumstances that might affect their credibility. For example, the distributions of suspected routes of transmission varied across 4 databases from studies on PLHIV in Korea [11,25]. Studies that utilized direct interviews and follow-up visits in hospital settings reported significantly fewer instances categorized as ‘others’ and ‘unknown’ compared to those derived from medical record reviews or surveillance data. Additionally, the reported ratios of same-sex to opposite-sex contact varied widely, ranging from 1.76:1.00 to 1.00:1.79. It was notable that the ratio of same-sex contact was much lower in the single-center study, which included PLHIV diagnosed earlier, compared to other studies. Moreover, the ratio increased over time in our study, aligning with this finding. Further research could be valuable in assessing the validity of self-reported information.

## DATA ACCESSIBILITY

Along with the national HIV/AIDS surveillance data, the cohort data are managed by the Division of HIV/AIDS Prevention at the KDCA. There is currently no established process for submitting applications to request data access or propose research. However, research ideas could be suggested through discussions with the Division and are subject to approval by the IRB of KDCA.

## Figures and Tables

**Table 1.** Categories and variables of the cohort data

Data categories	Variables
Incidence reports	
HIV diagnosis and report	Type of testing location, date of laboratory confirmation, date of report, province and city of report
Socio-demographic status	Birthdate, age at diagnosis, age at the time of report, biological sex, marital status
Epidemiological investigations	
HIV testing	Reason for testing
Route of transmission	High-risk experience for HIV exposure, suspected transmission route
Death reports	
HIV diagnosis and report	Date of laboratory-confirmation, type of testing location, date of report, province and city of report
Socio-demographic status	Birthdate, age at diagnosis, age at the time of report, biological sex, marital status
Information regarding death	Date of death, cause of death
Cause of death statistics	
Socio-demographic status	Marital status, highest educational level, occupation
Information regarding death	Date of death, location of death, cause of death (237 groups according to KCD-8)

HIV, human immunodeficiency virus; KCD-8, the 8th revision of Korean Standard Classification of Diseases.

**Table 2.** Socio-demographic characteristics of people living with HIV, reported in 1985-2020

Characteristics	Total reports (n=17,199)	Death reports (n=2,721)
Year of diagnosis		
1985-1990	119 (0.7)	70 (58.8)
1991-1995	376 (2.2)	196 (52.1)
1996-2000	757 (4.4)	321 (42.4)
2001-2005	2,529 (14.7)	694 (27.4)
2006-2010	3,799 (22.1)	796 (21.0)
2011-2015	4,814 (28.0)	464 (9.6)
2016-2020	4,805 (27.9)	180 (3.7)
Age at diagnosis (yr)		
19-29	5,194 (30.2)	298 (5.7)
30-39	4,636 (27.0)	613 (13.2)
40-49	3,606 (21.0)	713 (19.8)
50-59	2,428 (14.1)	578 (23.8)
60-69	992 (5.8)	349 (35.2)
≥70	343 (2.0)	170 (49.6)
Biological sex		
Female	1,125 (6.5)	184 (16.4)
Male	16,074 (93.5)	2,537 (15.8)
p-value^1^	0.61	
Marital status		
Not married	9,777 (56.8)	737 (7.5)
Married	4,501 (26.2)	1,014 (22.5)
Divorced	930 (5.4)	172 (18.5)
Cohabitation	276 (1.6)	39 (14.1)
Not available	1,715 (10.0)	759 (44.3)

Values are presented as number (%). HIV, human immunodeficiency virus.

1 Chi-square test.

**Table 3.** Epidemiological characteristics of people living with HIV, reported in 1985-2020

Characteristics	Year of diagnosis			
	Total (n=17,199)	1985-2000 (n=1,252)	2001-2010 (n=6,328)	2011-2020 (n=9,619)
Test reason				
General screening	2,199 (12.8)	<5	1,145	1,051
Illness	4,246 (24.7)	<5	1,464	2,781
Voluntary testing	2,344 (13.6)	0	654	1,690
Screening before hospitalization/surgery	2,218 (12.9)	<5	574	1,642
Partner's notification	112 (0.7)	0	36	76
Obligated routine testing	15 (0.1)	0	0	15
Blood donation	898 (5.2)	174	388	336
Others	799 (4.6)	6	631	162
Not available	4,368 (25.4)	1,066	1,436	1,866
Test location				
Medical facility	11,410 (66.3)	427	4,485	6,498
Public health center	4,025 (23.4)	515	1,344	2,166
Blood bank	1,281 (7.4)	173	460	648
Military facility	262 (1.5)	0	35	227
Correctional facility	40 (0.2)	0	<5	36
Quarantine	124 (0.7)	124	0	0
Private setting	44 (0.3)	0	0	44
Others	13 (0.1)	13	0	0
Transmission route				
Opposite-sex sexual contact	7,427 (43.2)	803	3,046	3,578
Same-sex sexual contact	5,598 (32.5)	308	2,181	3,109
Blood transfusion	32 (0.2)	25	7	0
Vertical transmission	0	0	0	0
Injection drug use	9 (0.1)	<5	<5	5
Not available	4,133 (24.0)	114	1,092	2,927

Values are presented as number (%) or number. HIV, human immunodeficiency virus.

**Table 4.** HIV and other top 20 causes of death of people living with HIV in Korea, reported in 1985-2020

Cause of death	KCD-8 code	Total casualties	1985-2000	2001-2010	2011-2020
HIV disease	B20-B24	1,817	122	783	912
Other and unspecified effects of external causes	T66-T78	129	12	51	66
Toxic effects of substances chiefly nonmedicinal as to source	T51-T65	54	5	21	28
Other ill-defined and unspecified causes of mortality	R99	47	6	12	29
Pneumonia, organism unspecified	J18	37	14	5	18
Tuberculosis	A15-A19	36	14	18	<5
Injuries to the head	S00-S09	35	5	11	19
Malignant neoplasm of liver and intrahepatic bile ducts	C22	31	<5	10	20
Malignant neoplasm of bronchus and lung	C34	30	<5	9	18
Acute myocardial infarction	I21	27	<5	8	15
Malignant neoplasm of stomach	C16	21	<5	7	10
Non-follicular lymphoma	C83	21	<5	<5	19
Fibrosis and cirrhosis of liver	K74	20	6	6	8
Injuries involving multiple body regions	T00-T07	18	<5	6	12
Cerebral infarction	I63	17	<5	7	8
Malignant neoplasm of pancreas	C25	16	<5	<5	10
Cardiac arrest	I46	13	<5	<5	9
Viral hepatitis	B15-B19	13	<5	7	6
Injuries to unspecified part of trunk, limb or body region	T08-T14	12	<5	<5	7
Other and unspecified types of non-Hodgkin lymphoma	C85	12	<5	<5	5
Other bacterial diseases	A30-A49	12	<5	<5	11

HIV, human immunodeficiency virus; KCD-8, 8th revision of Korea Standard Classification of Diseases.
